# Factors Contributing to Postoperative Radiotherapy Delays in Head and Neck Cancer: A Comprehensive Review

**DOI:** 10.7759/cureus.67240

**Published:** 2024-08-19

**Authors:** Abhijeet Sharma, Prasad Deshmukh, Shraddha Jain, Sagar S Gaurkar, Ayushi Sharma

**Affiliations:** 1 Otorhinolaryngology and Head and Neck Surgery, Jawaharlal Nehru Medical College, Datta Meghe Institute of Higher Education and Research, Wardha, IND; 2 Dental Sciences, People's College of Dental Science and Research Centre, Bhopal, IND

**Keywords:** cancer recurrence, multidisciplinary coordination, patient outcomes, treatment delays, postoperative radiotherapy, head and neck cancer

## Abstract

Head and neck cancer encompasses a diverse group of malignancies that pose significant challenges in their management due to their heterogeneity in clinical behavior and treatment response. Postoperative radiotherapy (PORT) is a critical component in the treatment regimen for head and neck cancer, aimed at reducing local recurrence and improving overall survival (OS). However, delays in the initiation of PORT can significantly compromise patient outcomes. This comprehensive review explores the factors contributing to such delays, categorizing them into patient-related, treatment-related, and systemic factors. Patient-related factors include health status, comorbidities, socioeconomic barriers, and psychological issues. Treatment-related factors involve surgical complications, complexities in treatment planning, and coordination challenges between surgical and radiation oncology teams. Systemic and institutional factors encompass hospital resources, staffing levels, administrative and insurance issues, and geographic barriers. The review also examined the impact of these delays on patient outcomes, highlighting the increased risk of recurrence and reduced survival rates. Strategies to mitigate delays are discussed, including improved preoperative and postoperative planning, enhanced multidisciplinary coordination, patient education, and systemic policy changes. Additionally, case studies and real-world examples of successful interventions are presented. Future directions for research and policy recommendations are also outlined, emphasizing the need for continued efforts to ensure timely PORT for head and neck cancer patients. This review aims to provide a comprehensive analysis that can inform clinical practice and policy, ultimately improving the standard of care and patient outcomes in head and neck cancer treatment.

## Introduction and background

Head and neck cancer encompasses diverse malignancies originating in the upper aerodigestive tract, including the oral cavity, pharynx, larynx, nasal cavity, and paranasal sinuses [1]. According to global cancer statistics, head and neck cancers account for approximately 4% of all cancers, with a significant prevalence in regions with high tobacco and alcohol consumption [2]. These cancers are notably heterogeneous in histopathology, clinical behavior, and response to treatment, which poses unique challenges in their management. The incidence of these cancers has been rising, especially in younger populations and those with human papillomavirus (HPV) infections, necessitating ongoing research and advancements in treatment protocols [3].

Postoperative radiotherapy (PORT) is a cornerstone in the management of head and neck cancer, aimed at eradicating microscopic residual disease, reducing the risk of local recurrence, and improving overall survival (OS) rates [4]. The timing of PORT is critical, with evidence suggesting that delays in initiating treatment can adversely affect patient outcomes. Studies have demonstrated that timely administration of radiotherapy within the recommended window significantly enhances therapeutic efficacy, minimizes recurrence, and improves survival rates. Conversely, delays in PORT have been associated with increased rates of local recurrence, disease progression, and poorer survival outcomes, underscoring the need for prompt and coordinated care [5].

This review aims to comprehensively explore the factors contributing to delays in PORT for head and neck cancer. By examining patient-related, treatment-related, and systemic factors, we seek to highlight the multifaceted nature of these delays and their impact on patient outcomes. Additionally, this review will discuss strategies to mitigate these delays, supported by case studies and emerging research. The goal is to provide a detailed analysis that can inform healthcare providers, researchers, and policymakers, ultimately enhancing the standard of care for head and neck cancer patients. This review aims to identify critical areas for intervention and improvement, ensuring that all patients receive timely and effective PORT.

## Review

Clinical importance of PORT

PORT is pivotal in managing head and neck cancer, particularly in reducing recurrence rates and improving OS. Its clinical significance is emphasized by the high risk of local-regional recurrence in patients with squamous cell carcinoma of the head and neck following surgical resection [4]. PORT has been demonstrated to effectively mitigate this risk, which is crucial since locoregional failure is a significant cause of mortality in these patients. Research shows that patients receiving PORT have better local-regional control rates compared to those who do not. Specifically, continuous-course radiotherapy has been associated with superior outcomes compared to split-course regimens, underscoring the necessity of PORT in managing high-risk patients to prevent recurrence [6]. The impact of PORT on OS is also notable. While some studies suggest that despite reducing local-regional recurrence, PORT may increase the risk of distant metastases, leading to debates about its OS benefits, many clinicians advocate for its use in high-risk populations [7]. Evidence indicates that patients undergoing continuous-course PORT have significantly better survival rates than those receiving split-course therapy. This highlights the importance of treatment timing and regimen in enhancing survival outcomes, reinforcing the role of PORT as a critical intervention in the postoperative setting [7]. Guidelines for initiating PORT recommend starting treatment as soon as possible after surgery to minimize delays. Ideally, the overall treatment time from surgery to the start of radiotherapy should be, at most, six weeks, with the entire treatment course completed within 10 weeks to optimize patient outcomes [8]. Accelerated radiotherapy schedules have been proposed to enhance treatment efficacy, particularly for high-risk patients, by reducing the interval between surgery and the initiation of radiation therapy. This approach aims to decrease overall treatment time, improving local control and survival rates [8].

Overview of PORT

PORT is a vital component of cancer treatment, particularly following the surgical resection of tumors. It involves applying radiation therapy after surgery to eliminate any residual microscopic disease, thereby enhancing local disease control and improving OS rates, especially in high-risk cancer patients [9]. The primary objectives of PORT are to target and eradicate remaining cancer cells at the surgical site, thereby reducing the risk of locoregional recurrence. By achieving local control, PORT significantly contributes to long-term survival, particularly in locally advanced cancers, where a multimodal treatment approach, including chemotherapy or targeted therapies, is often necessary [10]. The protocols for administering PORT can vary based on several factors, including the type of cancer, the extent of the disease, and individual patient characteristics. Commonly employed radiation techniques include intensity-modulated radiotherapy (IMRT) and three-dimensional conformal radiation therapy (3D-CRT), both of which optimize dose delivery while minimizing exposure to surrounding healthy tissues [11]. Treatment planning involves detailed imaging studies, such as CT, MRI, and PET scans, to accurately define target volumes, including the tumor bed and affected lymph nodes. The radiation dose is typically delivered in fractions over several weeks, with schedules adjusted according to the patient's overall health and tumor characteristics. Additionally, patients receiving PORT are closely monitored for side effects and treatment response, with follow-up imaging and clinical assessments to evaluate for recurrence [12]. Various factors influence PORT's expected outcomes, including tumor type, stage, and individual patient characteristics. A key benchmark for assessing the effectiveness of PORT is its impact on recurrence rates. Research has shown that PORT significantly reduces locoregional recurrence rates in high-risk patients, particularly in head and neck cancers, where it can decrease the risk of local failure, a significant cause of mortality. Furthermore, studies indicate that PORT can improve OS rates, especially in patients with advanced disease [13]. For instance, integrating PORT into treatment protocols for head and neck cancers has been associated with better survival outcomes than surgery alone. However, while PORT can enhance survival and disease control, it may also lead to acute and chronic side effects impacting the patient's quality of life. Ongoing research aims to balance the benefits of PORT with the effective management of these side effects, ensuring that patients maintain a good quality of life post-treatment [14].

Factors contributing to delays

Patient-Related Factors

Patient-related factors play a significant role in the timely initiation of PORT for individuals diagnosed with head and neck cancer. One of the primary considerations is the patient’s health status and comorbidities. Patients with multiple comorbid conditions, such as diabetes or cardiovascular disease, often face complications that can prolong their recovery time after surgery [15]. Additionally, an individual’s overall health can significantly impact their recovery rate. Those with poorer baseline health may require extended postoperative monitoring and care, which can delay the start of necessary radiotherapy [15]. Recovery from surgery is another critical factor influencing the timeline for PORT. The extent of surgical intervention varies among patients, and those who experience prolonged recovery due to surgical complications may find themselves unable to begin radiotherapy as scheduled. Furthermore, functional status plays a vital role; patients who suffer from significant impairments, such as difficulty swallowing or persistent pain, may need additional time for rehabilitation before they can tolerate the rigors of radiotherapy [16]. Socioeconomic status and access to care are also crucial determinants of treatment delays. Patients from lower socioeconomic backgrounds often encounter financial barriers that hinder their ability to pursue timely treatment. High out-of-pocket costs and inadequate insurance coverage can deter patients from adhering to treatment schedules. Additionally, transportation issues can pose significant challenges, particularly for those living in rural or underserved areas, further complicating their access to specialized care [17]. Lastly, psychological factors and patient compliance are essential to consider. Mental health issues, such as anxiety and depression, can profoundly affect a patient’s motivation and willingness to adhere to treatment protocols. Patients experiencing psychological distress may delay seeking further treatment or struggle to commit to scheduled appointments. Moreover, compliance with medical advice and follow-up care is critical; factors influencing a patient’s adherence include their understanding of the treatment plan, the perceived severity of their illness, and the presence of a supportive social network [18].

Treatment-Related Factors

Delays in PORT for head and neck cancer can be significantly influenced by treatment-related factors. One of the primary contributors is surgical complications. Patients who experience complications during or after surgery often require extended recovery times, which can push back the initiation of radiotherapy [19]. Common complications include infections, bleeding, and issues related to wound healing. These complications not only prolong hospital stays but also create a gap between surgery and the start of PORT, ultimately impacting the patient’s prognosis [19]. Another critical factor is the complexity of treatment planning. Head and neck cancer treatment typically requires a multidisciplinary approach involving various specialties, including surgical oncology, radiation oncology, and medical oncology [20]. The complexity of developing an effective treatment regimen tailored to each patient's unique anatomy and cancer characteristics can be time-consuming. This intricate planning process may delay the overall treatment timeline, mainly if there are disagreements or uncertainties among the involved specialists [20]. Coordination between surgical and radiation oncology teams is also vital in preventing delays. Effective communication is crucial for timely treatment; any lapses can result in scheduling issues for radiotherapy sessions. Regular interdisciplinary meetings are essential for better coordination, but if these meetings are infrequent or poorly organized, they can lead to delays in decision-making and treatment initiation. Improved collaboration among healthcare providers can help streamline the transition from surgery to radiotherapy [21]. Finally, the availability of radiotherapy slots and equipment plays a significant role in determining the timing of PORT. Resource limitations, such as the high demand for radiotherapy services in specific healthcare settings, can delay scheduling. Additionally, equipment downtime due to maintenance or breakdowns can exacerbate these delays, especially in facilities that need backup machines or sufficient resources to manage patient loads effectively [22]. Addressing these treatment-related factors is essential for minimizing delays in PORT for head and neck cancer patients. By improving surgical outcomes, enhancing communication and coordination among healthcare teams, and optimizing the use of radiotherapy resources, healthcare providers can improve the efficiency of cancer treatment pathways and ultimately enhance patient outcomes [5].

Systemic and Institutional Factors

The factors contributing to delays in initiating PORT for head and neck cancer patients are multifaceted, encompassing demographic, socioeconomic, clinical, and systemic/institutional elements [23]. Demographic and socioeconomic factors, such as race, ethnicity, insurance status, and income level, play a significant role. Studies have shown that Black and Hispanic patients face higher odds of experiencing PORT delays, with odds ratios (OR) of 1.46 and 1.37, respectively. Patients with Medicaid or those who are uninsured face even more significant risks, with an OR of 2.01, highlighting the disparities in access to timely care. Additionally, lower income is associated with a higher likelihood of delayed treatment, with an OR of 1.38 [24]. Clinical factors, including postoperative complications, health literacy, and geographic location, also contribute to delays in initiating PORT. Extended hospital stays (greater than seven days) significantly increase the risk of delays, with an OR of 2.92, as do 30-day hospital readmissions following surgery (OR, 1.37). Lower levels of written health literacy correlate with delays in initiating PORT, underscoring the importance of patient education and support in navigating treatment pathways. Patients residing in rural areas may face additional barriers, such as limited access to specialized care facilities [24].

**Figure 1:**
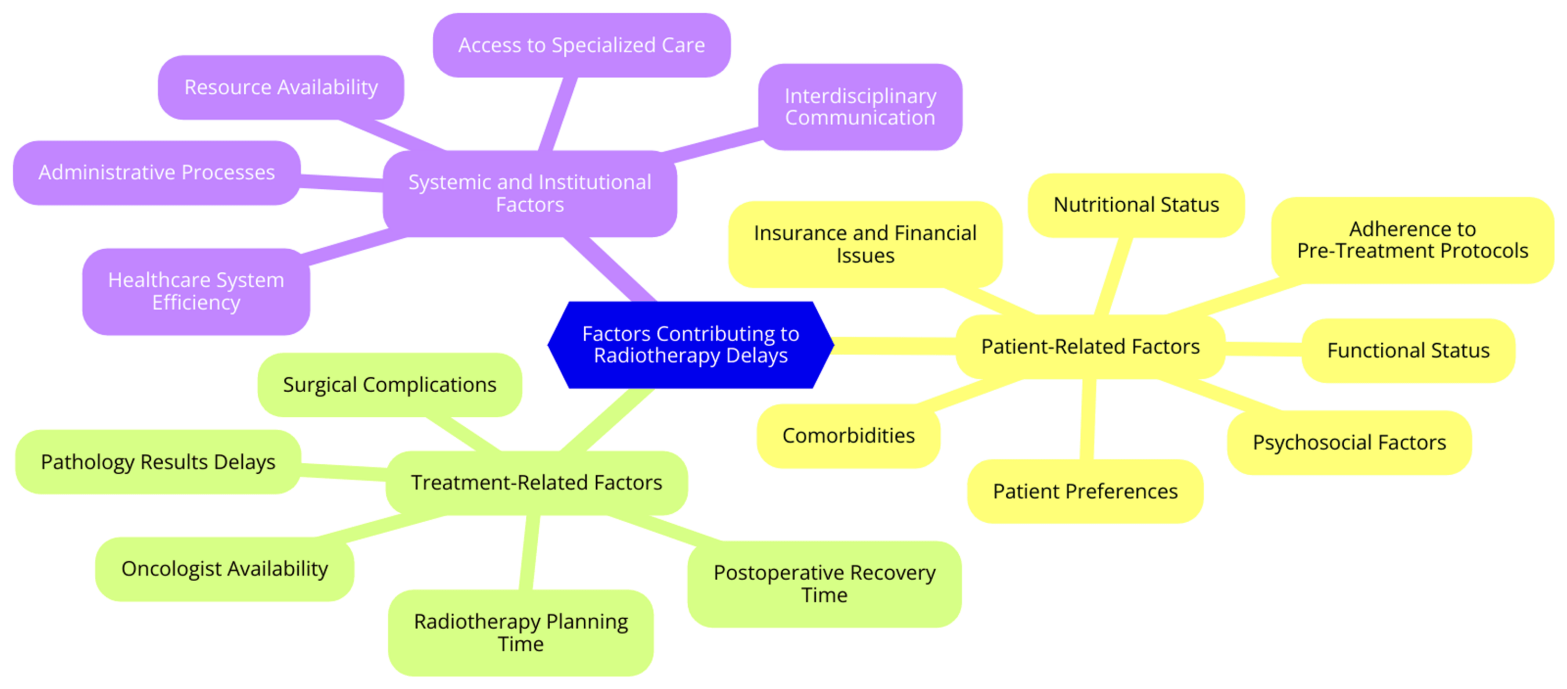
Factors contributing to postoperative radiotherapy delays in head and neck cancer Image credit: Dr. Abhijit Sharma

Systemic and institutional factors, such as hospital and clinic resources, staffing levels and expertise, administrative and insurance issues, and geographic and logistical barriers, also play a crucial role in treatment delays [25-26]. Shortages of radiation oncologists, medical physicists, and radiation therapists and a lack of specialized expertise can prolong treatment initiation. Complex administrative processes, prior authorization requirements, and insurance-related delays can significantly contribute to treatment delays. Patients living in remote areas or facing transportation challenges may experience difficulties accessing specialized cancer centers, leading to delays in initiating PORT [26]. By addressing these multifaceted factors, healthcare systems can enhance the quality of care and minimize delays in initiating PORT for head and neck cancer patients. Strategies should focus on improving health literacy, streamlining care coordination, addressing socioeconomic barriers, optimizing resource allocation, improving staffing levels and expertise, and enhancing administrative processes [27]. By implementing targeted interventions and addressing the systemic and institutional challenges, healthcare providers can ensure timely and equitable access to PORT for head and neck cancer patients, ultimately improving survival outcomes [27]. Factors contributing to PORT delays in head and neck cancer are shown in Figure 1.

Impact of delays on patient outcomes

Delays in treatment initiation for head and neck cancer have significant implications for patient outcomes, impacting both the effectiveness of therapy and overall quality of life. One of the most critical consequences of delayed PORT is the increased risk of local recurrence. Research indicates that starting radiation therapy more than six weeks after surgery can lead to higher rates of recurrence, particularly in patients with advanced disease stages. The hazard of recurrence is notably more significant when treatment initiation exceeds recommended timeframes, underscoring the importance of timely intervention [28]. In addition to the risk of local recurrence, delays in treatment are associated with reduced OS rates. Numerous studies have demonstrated a strong correlation between treatment delays and worse survival outcomes in head and neck cancer patients. Specifically, mortality rates tend to increase significantly when treatment is postponed beyond 60 days. This effect is especially pronounced in patients with earlier-stage cancers, where delays can lead to stage migration and subsequently poorer prognoses. The urgency of timely treatment cannot be overstated, as it is crucial in improving long-term survival [29]. Furthermore, delays in treatment can result in more aggressive disease progression. Head and neck cancers are typically fast-growing, and any delay in treatment can allow the cancer to advance to a more severe stage. This progression often necessitates more aggressive treatment approaches, which can increase morbidity and complicate the overall treatment process. As the disease becomes more advanced, the challenges associated with treatment escalation can lead to poorer patient outcomes in terms of survival and quality of life [30]. Quality-of-life considerations are also significantly impacted by treatment delays. Patients awaiting treatment may experience heightened anxiety, pain, and other symptoms associated with advanced disease. The psychological burden of waiting for treatment can take a toll on patients’ emotional well-being, leading to feelings of helplessness and fear regarding disease progression. This decline in overall quality of life can further complicate the patient’s experience and may affect their willingness to adhere to treatment protocols [31].

Strategies to mitigate delays

To mitigate delays in PORT for head and neck cancer, a multifaceted approach addressing various aspects of the treatment process is essential. One effective strategy is to improve preoperative and postoperative planning. This can include scheduling early radiation oncology consultations before surgery, which helps streamline the transition to PORT and minimizes delays [19]. Additionally, conducting preoperative risk assessments allows healthcare providers to identify high-risk patients for complications, facilitating proactive planning and resource allocation. Implementing standardized postoperative care pathways based on evidence-based protocols can further reduce variability in care and optimize recovery timelines [19]. Enhancing coordination and communication between multidisciplinary teams is another crucial strategy. Regular meetings among surgical, radiation oncology, and medical oncology teams can foster better collaboration and ensure all parties are aligned on the patient’s treatment plan [32]. Employing dedicated care, coordinators can facilitate patient transitions and ensure timely referrals while integrating treatment planning and scheduling systems within electronic health records (EHR), which can improve data sharing and streamline the referral process [32]. Patient education and support systems also play a vital role in reducing delays. Providing clear information to patients about the importance of timely initiation of PORT can help set appropriate expectations and encourage adherence to the treatment plan [33]. Additionally, offering psychosocial support services to address emotional and practical concerns can help mitigate barriers to timely treatment. Resources such as transportation and lodging assistance for patients facing logistical challenges can ensure they can attend their scheduled appointments [33]. Lastly, implementing policy and systemic changes at the institutional level is critical for long-term improvement. Quality improvement initiatives that include regular audits and feedback mechanisms can monitor PORT initiation timelines and identify areas for enhancement. Developing and enforcing clear institutional policies regarding the timely initiation of PORT can help set expectations and hold teams accountable [34]. Furthermore, ensuring adequate staffing, equipment, and infrastructure to meet the demand for PORT can alleviate delays caused by capacity constraints. By adopting these comprehensive strategies, healthcare institutions can significantly reduce delays in PORT for head and neck cancer patients, ultimately improving patient outcomes and quality of life [34].

Future directions and research

Several emerging technologies significantly promise to reduce delays in initiating PORT for head and neck cancer patients. Artificial intelligence (AI) and machine learning are among the most impactful advancements. These technologies can analyze vast amounts of patient data to predict which individuals are at the highest risk of experiencing delays based on demographic, clinical, and social factors. By identifying at-risk patients early, healthcare providers can implement proactive interventions that address specific barriers, ensuring timely treatment initiation [35]. Digital health tools also present a valuable opportunity to enhance patient engagement and streamline the transition from surgery to radiotherapy. Patient-facing applications and online portals can offer educational resources, support, and reminders to help patients navigate the complexities of recovery and prepare for radiation therapy [36]. This increased engagement may lead to improved adherence to treatment plans and reduced delays. Additionally, telehealth services can facilitate remote monitoring and virtual consultations, allowing healthcare providers to follow patients post-surgery and identify potential issues early on. This proactive approach can significantly decrease the likelihood of unplanned hospitalizations, often contributing to delays in PORT [36]. Moreover, precision scheduling powered by advanced analytics can optimize the timing of PORT based on individual patient needs provider availability, and resource constraints. By improving scheduling efficiency, healthcare systems can minimize delays and enhance overall patient care [37].

To effectively address the issue of PORT delays, several critical areas for future research and clinical trials should be prioritized. Prospective studies are needed to validate risk prediction models that identify patients likely to experience delays and test targeted interventions to mitigate these delays in high-risk populations. Additionally, clinical trials evaluating the effectiveness of digital health tools and telehealth approaches can provide valuable insights into how these technologies support patients in the postoperative period and prepare them for radiation therapy [38]. Comparative effectiveness research is also crucial in determining the optimal timing and sequencing of surgical interventions, PORT, and systemic therapies. Understanding the best approaches to multimodal treatment will help improve patient outcomes. Furthermore, implementation science studies should identify barriers and facilitators to timely PORT initiation at various levels, including provider, health system, and policy [39]. Policymakers should consider several strategic recommendations to enhance outcomes for head and neck cancer patients. Expanding insurance coverage and improving access to care, particularly for underserved populations at a higher risk of experiencing delays, is essential. Additionally, incentivizing health systems to invest in technologies and processes that support timely PORT initiation, such as predictive analytics, digital patient engagement tools, and precision scheduling, can drive improvements in care delivery [40]. Developing national quality metrics and benchmarks for PORT timeliness is another important step. Such measures can foster accountability and encourage continuous improvement within healthcare systems. Finally, funding research initiatives to develop and validate innovative approaches to reducing PORT delays and optimizing treatment sequencing is critical for advancing the field [41].

## Conclusions

Timely PORT is essential for optimizing outcomes in patients with head and neck cancer. This review has highlighted the complex interplay of patient-related, treatment-related, and systemic factors contributing to delays in initiating radiotherapy. These delays can significantly impact local recurrence rates, OS, and patient quality of life, underscoring the need for coordinated and efficient healthcare delivery. By identifying and addressing these barriers through improved preoperative and postoperative planning, enhanced communication among multidisciplinary teams, and systemic policy changes, healthcare providers can better support patients receiving timely treatment. Emerging technologies and ongoing research also promise to mitigate delays and improve patient outcomes. Ultimately, addressing the multifaceted causes of delays in PORT is crucial for advancing the standard of care and ensuring that patients with head and neck cancer achieve the best possible outcomes. This comprehensive review serves as a call to action for clinicians, researchers, and policymakers to prioritize and implement strategies that reduce delays and enhance the quality of care for these patients.
